# Mitochondria bridge HIF signaling and ferroptosis blockage in acute kidney injury

**DOI:** 10.1038/s41419-022-04770-4

**Published:** 2022-04-06

**Authors:** Wenju Li, Zhidan Xiang, Yuexian Xing, Shen Li, Shaolin Shi

**Affiliations:** 1 National Clinical Research Center for Kidney Diseases, Jinling Hospital, Nanjing University School of Medicine, Nanjing, China; 2 Department of Endocrinology, The Third Affiliated Hospital of Soochow University, Changzhou, China

**Keywords:** Cell biology, Kidney diseases

## Abstract

Ferroptosis, a form of regulated cell death, plays an important role in acute kidney injury (AKI). Previous studies have shown that prolyl hydroxylase domain protein (PHD) inhibitors that activate HIF signaling provide strong protection against AKI, which is characterized by marked cell death. However, the relationship between PHD inhibition/HIF signaling and ferroptosis in AKI has not been elucidated. Here, we review recent studies to explore the issue. First, we will review the literature concerning the functions of HIF in promoting mitophagy, suppressing mitochondrial respiration and modulating redox homeostasis. Second, we will describe the current understanding of ferroptosis and its role in AKI, particularly from the perspective of mitochondrial dysfunction. Finally, we will discuss the possibility that mitochondria link PHD inhibition/HIF signaling and ferroptosis in AKI. In conclusion, we propose that HIF may protect renal cells against ferroptosis in AKI by reducing mitochondrial oxidative stress and damage.

## Facts

- Ferroptosis plays an important role in the development of AKI.
- Activation of HIF signaling provides protection against AKI.
- Mitochondrial ROS facilitate ferroptosis in many cases.
- HIF signaling regulates multiple mitochondrial activities, including mitochondrial oxidative capacity, redox homeostasis and autophagy.

## Open questions

- Can HIF signaling alleviate ferroptosis in AKI with different etiologies?
- Is mitochondrial pathway decisive for HIF-offered protection in situations like AKI, given that HIF signaling has extensive downstream effects?
- Would PHD inhibitors be effective in AKI prevention and treatment clinically?

## Introduction

Acute kidney injury (AKI) is a common disease with high morbidity and mortality. It is characterized by the rapid loss of kidney function as determined by the accumulation of creatinine in blood and a decrease in urine output. There are no particularly effective treatments for AKI. Patients have an increased risk of subsequent chronic kidney disease after they recover from AKI. Pathologically, AKI is characterized by tubular structural damage, cell death, and inflammation.

AKI primarily involves renal tubules. Tubular cells are highly energy-demanding, which makes them prone to damage induced by hypoxia and mitochondrial dysfunction [1, 2]. The key transcription factor mediating hypoxic adaptation, hypoxia-inducible factor (HIF), is commonly involved in the pathophysiology of AKI, whether or not it is caused by ischemia [2]. On the other hand, due to the essential role of mitochondria in the pathogenesis of AKI, drugs that target mitochondria may be promising in AKI treatment. Here, we review the latest studies on the regulation of mitochondrial function by HIF signaling and further associate this regulation with ferroptosis, which has been identified as an essential form of cell death in AKI in recent years.

## HIF as a pivotal regulator of mitochondria-related biological processes

Mitochondria are the center of cellular energy production and consume most of the oxygen in the cell. Most eukaryotic cells rely on oxygen for respiratory energy production, while in hypoxia, cellular metabolism is forced to be reprogrammed, which manifests as a boost in anaerobic glycolysis and suppression of mitochondrial activity [3]. HIF transcriptionally orchestrates the metabolic switch and is well known for its role in regulating the glycolytic machinery. However, studies have increasingly illustrated the important role of HIF in mitochondrial functions, biogenesis, turnover, and redox homeostasis.

### HIF transduces transcriptional responses to hypoxia

Oxygen supply is the basic survival need for most eukaryotic organisms to fuel cellular respiration. Oxygen delivery function has evolved to a complex system covering respiratory and circulatory tissues in mammals, which could be inevitably influenced by multiple physiological and pathological events. Exquisite mechanisms that maintain oxygen homeostasis can operate over a range of oxygen concentrations, as many tissues function physiologically at different oxygen levels and in response to temporal and chronic challenges [4]. Control of gene expression by oxygen levels has an essential role in this oxygen sensing system. Erythropoietin (EPO) was first discovered to stimulate erythrocyte production in response to low blood oxygen, while studies of its transcriptional regulation have led to the identification of HIF and further confirmation of HIF’s extensive regulatory effects across cell types [5, 6].

HIF is known as the key transcription factor mediating adaptation to hypoxia [7]. It is a protein heterodimer composed of a hypoxia­sensitive α subunit (HIF­α) and a constitutively expressed β subunit (HIF­β). Three isoforms of HIF-α and two of HIF­β (arylhydrocarbon receptor nuclear translocator (ARNT/HIF-1β) and ARNT2) have been identified in most vertebrate species. HIF-1β is abundantly expressed in most cells, and the HIF­1α, HIF­2α and HIF­3α complexes with HIF­1β are termed HIF­1, HIF­2, and HIF­3 [8]. HIF-1 and HIF-2 direct largely distinct transcriptional systems in response to hypoxia based on their cell­type-specific expression and DNA binding selectivity [9, 10]. The function of HIF-3α is less well understood, partly due to the existence of multiple HIF-3α variants originating from different transcription initiation sites and alternative splicing. Nevertheless, some variants of HIF-3α were revealed to have negative feedback effects on HIF-1/2 [11].

**Fig. 1:**
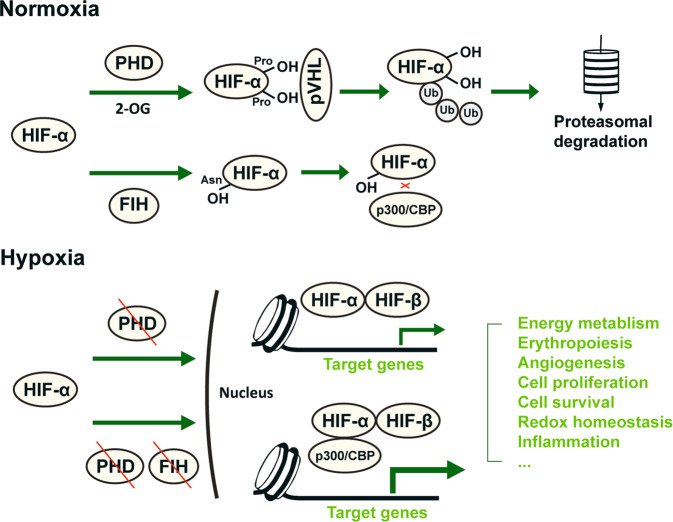
Schematic illustration of the posttranslational regulation and function of HIF. HIF is the principal transcription factor mediating adaptation to oxygen tension, regulating a wide variety of biological processes, such as energy metabolism, erythropoiesis, angiogenesis, redox homeostasis, inflammation response, cell proliferation and survival. It is a heterodimer composed of a hypoxia­sensitive HIF­α subunit and a constitutively expressed HIF­β subunit. HIF­1α and HIF­2α are constantly hydroxylated by PHD1-3 at specific prolyl residues in an oxygen- and 2-OG-dependent process and targeted for proteasomal degradation, whereas during hypoxia, they dimerize with HIF­β to bind to DNA sequences and regulate gene expression. In more severe hypoxia, asparaginyl hydroxylation of HIF-α by FIH is inhibited, and increased interaction of HIF with the transcriptional coactivators p300 and CBP further promotes target gene expression. PHD prolyl hydroxylase domain protein. 2-OG oxoglutarate. FIH factor-inhibiting HIF. CBP CREB-binding protein.

The oxygen sensitivity of HIF­1α and HIF­2α is achieved by regulatory enzymes that catalyze the hydroxylation of their specific prolyl and asparaginyl residues, as reviewed in detail elsewhere [4] (Fig. 1). In brief, prolyl hydroxylation by prolyl hydroxylase domain (PHD) enzymes is highly dependent on the oxygen concentration. Hydroxyprolyl is recognized by von Hippel-Lindau suppressor, which is a subunit of an E3 ubiquitin ligase complex that leads to rapid proteasomal degradation of HIF­α. In hypoxia, the activity of PHDs is restricted, and HIF-α dimerizes with HIF-1β to form transcriptionally active complexes capable of binding with high affinity to DNA sequences at hypoxia response elements (HREs) and thus modulating gene transcription. Another enzyme named factor-inhibiting HIF (FIH) hydroxylates an asparagine residue in the C­terminal domain of HIF­α protein, preventing its interaction with the transcriptional coactivators p300 and CREB-binding protein and therefore partially hindering the activity of HIF as a transcription factor [12, 13]. FIH activity is inhibited at lower oxygen concentrations than PHDs, which thus provides a hierarchic adaptive mechanism to oxygen tension [4].

Subsequently, HIF regulates the expression of hundreds of target genes involved in various biological processes, such as metabolism, erythropoiesis, angiogenesis, the cell cycle and survival [7, 10]. The major result of hypoxia is a dramatic reduction in energy production. At the cellular level, HIF primarily mediates metabolic reprogramming, facilitating anaerobic ATP production from glycolysis by increasing the expression of glucose transporters, glycolytic enzymes, and lactate dehydrogenase A (LDHA) [7, 14]. Aerobic mitochondrial metabolism is also substantially modulated by HIF, which will be discussed in the following section. Moreover, HIF stimulates lipid storage and inhibits lipid catabolism both in mitochondria and peroxisomes [14, 15].

As PHDs are 2-oxoglutarate (2-OG, α-ketoglutarate)-dependent dioxygenases, chemical 2-OG analogs can be used for PHD inhibition and HIF-α stabilization and can serve as leads for drug discovery targeting the HIF/EPO axis. In recent years, several PHD inhibitors have been shown to be effective in renal anemia treatment in clinical trials, in which roxadustat (FG-4592) has been approved for clinical use in some countries based on its overall effectiveness [16].

### HIF downregulates mitochondrial respiration for metabolic adaptation

**Fig. 2:**
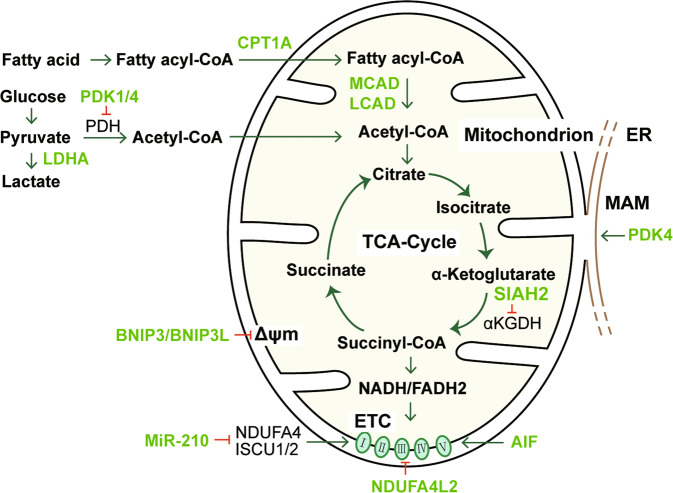
HIF-mediated regulation of mitochondrial metabolism. For hypoxic adaptation, HIF transcriptionally regulates the expression of a subset of genes to reduce mitochondrial metabolism and oxygen consumption. The expression of these genes influences the core functions of mitochondria by affecting TCA cycle substrate availability, Δψm, TCA cycle operation and respiratory chain function. The molecules directly regulated by HIF are shown in lime color. ER endoplasmic reticulum. MAM mitochondria-associated ER membrane. ETC electron transport chain. TCA cycle tricarboxylic acid cycle. Δψm mitochondrial membrane potential.

Oxidative ATP production from mitochondrial oxidative phosphorylation (OXPHOS) is an intricate process involving electron transport chain (ETC)-mediated hydrogen transfer and electron transfer. The tricarboxylic acid (TCA) cycle in the mitochondrial matrix catabolizes acetyl-CoA through a series of enzymatic reactions, generating the reducing equivalents NADH and FADH_2_ that provide electrons to the ETC. By regulating the expression of a subset of proteins, HIF reduces TCA cycle substrate availability, thereby decreasing mitochondrial respiration rates, oxygen consumption, and ROS production (Fig. 2).

Acetyl-CoA can be generated from oxidative decarboxylation of pyruvate from glycolysis, and β-oxidation of fatty acids. Both glycolysis and β-oxidation are regulated by HIF. HIF increases the expression of pyruvate dehydrogenase kinase (PDK) 1 and PDK4, which phosphorylate and thus inactivate the mitochondrial enzyme pyruvate dehydrogenase (PDH), resulting in a reduction in pyruvate-derived acetyl-CoA generation [17, 18]. PDK1 activation and aerobic glycolysis promotion by HIF-1 were linked to lowered levels of oxygen consumption and ROS generation, both in vitro and in vivo [19–21]. LDHA is also upregulated by HIF and diverts pyruvate away from conversion to acetyl-CoA [22]. On the other hand, HIF-1 and HIF-2 downregulate the expression of carnitine palmitoyltransferase 1A, which acts to facilitate the transport of long-chain fatty acids into mitochondria [23], as well as two related enzymes, namely, acyl-CoA dehydrogenases MCAD and LCAD, which catalyze fatty acid β-oxidation in the mitochondria [24]. Downregulation of these genes by HIF further lowers the levels of oxidative phosphorylation, oxygen consumption and ROS production in cells under hypoxia. These processes may also involve other HIF-regulated transcription factors, e.g., PGC-1α, PPARα, and PPARγ [25, 26].

SIAH2 is an E3 ubiquitin-protein ligase that mediates ubiquitination and proteolysis of the E1 subunit of α-ketoglutarate dehydrogenase (αKGDH), a key enzyme in the TCA cycle, to maintain its activity at an optimal level. Upon hypoxia, HIF-1 promotes SIAH2 activity and thus dramatically reduces αKGDH enzymatic activity, resulting in a decrease in mitochondrial oxygen consumption and glutamine oxidation [27]. Recently, miR-210 was shown to be markedly induced in hypoxia by HIF at the transcriptional level in proximal tubule cells [28]. miR-210 induces inner mitochondrial membrane damage by targeting ISCU1/2 and NDUFA4. ISCU1/2 are scaffold proteins for the de novo synthesis of iron–sulfur (Fe–S) clusters within mitochondria, which generally function as the active center of catalysts or participate in electron transfer. NDUFA4 is a component of ETC complex I and complex IV. Therefore, the HIF/miR-210 pathway can reduce mitochondrial respiration in hypoxia by acting on these components in the respiratory chain [28, 29]. Interestingly, HIF-1 induces the expression of NDUFA4 mitochondrial complex associated like 2 (NDUFA4L2), a protein that is highly similar in sequence with NDUFA4; however, NDUFA4L2 induction contributes to a decrease in complex I activity, mitochondrial oxygen consumption and ROS production [30]. Apoptosis-inducing factor (AIF) is an evolutionarily conserved mitochondrial intermembrane flavoprotein. AIF plays a dual role in regulating cell survival and apoptosis: its translocation to the nucleus causes caspase-independent apoptosis in response to death stimuli, while in the mitochondria, AIF maintains OXPHOS function via posttranscriptional regulation of respiratory chain complexes through its NADH oxidoreductase activity [31, 32]. It was shown that binding of HIF-1 in the promoter significantly repressed rather than activated AIF expression [33]. As AIF deficiency potently compromises OXPHOS [32, 34], it is reasonable to speculate that AIF might be another target by which HIF downregulates mitochondrial respiration.

The mitochondrial membrane potential (Δψm) is essential for ATP production from OXPHOS, and its loss represents impairment of mitochondrial function. One common cause of Δψm collapse is the opening of the mitochondrial permeability transition pore (mPTP). mPTP opening permits communication between the mitochondrial matrix and the cytoplasm, likely resulting in cell death [35]. HIF seems to have paradoxical influences on Δψm maintenance. On the one hand, HIF-1 inhibits mPTP opening to stabilize the Δψm and decreases mitochondrial oxidative stress following ischemia–reperfusion injury (IRI) in the heart [21]. In cultured renal tubular cells, HIF-1 reversed hypoxia-induced mitochondrial Δψm loss and ROS generation by heme oxygenase-1 (HO-1) induction [36]. HIF-1α associated with the outer mitochondrial membrane (OMM) protects the integrity of ΔΨm and prevents apoptosis by directly regulating voltage-dependent anion channel 1 (VDAC1) [37]. On the other hand, in normoxia, HIF-1 activation was found to cause Δψm loss and mitochondrial dysfunction [19]; however, the detailed mechanism is not clear, although several genes transcriptionally regulated by HIF have been reported to be capable of inducing Δψm loss. In hypoxia, HIF strongly upregulates the expression of the mitophagy receptors BNIP3 and BNIP3L [15, 38]. BNIP3L can promote Ca^2+^ release from the endoplasmic reticulum (ER), and the subsequent mitochondrial Ca^2+^ accumulation could induce mitochondrial permeability transition and thus Δψm collapse [39]. BNIP3 has been shown to exert similar functions [40, 41]. As mentioned above, PDK4 phosphorylates and thus inhibits the PDH complex, resulting in a decrease in glucose metabolism. Recently, it was found that PDK4 not only drives metabolic reprogramming toward glycolysis but also regulates the formation of mitochondria-associated ER membrane and subsequent Ca^2+^ transport, reducing Δψm and impairing mitochondrial respiratory capacity [42, 43]. In cisplatin-induced AKI, PDK4 knockout attenuated the disruption of mitochondrial morphology and the suppression of Δψm and the mitochondrial oxygen consumption rate [44]. The opposite effects of HIF on Δψm might be attributed to a variety of factors, including the property and strength of injurious stimuli, the level of HIF activity, and the cell type. Nevertheless, the differential effects of HIF may reflect the complex roles of HIF in cellular stress responses by diverse mechanisms. We speculate that HIF may promote a reversible Δψm decrease at a normal level of activation by inhibiting mitochondrial respiration capacity. This action of HIF could protect the cells from devastating oxidative stress and irreversible mitochondrial injury in hypoxia.

Notably, HIF-1α was reported to translocate to the OMM, where it protects against oxidative stress-induced cell death by a mechanism independent of its nuclear transcriptional regulation [45]. In this scenario, mitochondrial HIF attenuates ROS production, mitochondrial membrane potential collapsing, and mitochondrial DNA-encoded mRNA expression in response to hypoxia or H_2_O_2_ treatment. However, the effects cannot be fully explained by the previously reported mechanism of HIF-1α/mortalin/VDAC1 [37, 45]. Further understanding of the role and underlying mechanism of OMM-linked HIF is important, as OMM-translocated HIF may provide cells with immediate protection against injurious stimuli, potentially having therapeutic significance.

### HIF promotes mitophagy by regulating essential mitophagy-inducing factors

As the cellular energy center, mitochondria are highly dynamic and in constant turnover for quality control. In the selective autophagy process of mitochondria, referred to as mitophagy, cells remove senescent and damaged mitochondria, which are prone to produce more ROS and provoke apoptosis [46]. Furthermore, in addition to the quality control mechanism in steady-state conditions, mitophagy is needed to remove superfluous mitochondria in response to homeostatic perturbations. Dysfunctional or insufficient mitophagy has been proven to be responsible for various pathological situations, such as IRI in different organs [47, 48], contrast-induced AKI [49], diabetic cardiomyopathy [50], Alzheimer’s disease [51] and aging [52]. Interestingly, HIF has been demonstrated to induce mitophagy in hypoxia, and as an adaptive response, this regulation is necessary to prevent increased levels of ROS and cell death [53].

Mitophagy takes place in two distinct pathways that are receptor-dependent or ubiquitin-dependent. In receptor-dependent mitophagy, mitochondria are tagged with degradation signals on proteins (receptors) at the OMM and are thus recognized and connected to the autophagosomal membrane [17]. A number of OMM-localized proteins, including BNIP3, BNIP3L (NIX), FUNDC1 and AMBRA1, can serve as receptors and are directly linked with LC3B [54]. HIF-1-dependent BNIP3 expression was proven to induce mitophagy in prolonged hypoxia [53]. HIF-1 enhances the expression of BNIP3 and its closely related protein BNIP3L by directly binding to HREs on their promoters [53, 55, 56]. On the OMM, both BNIP3 and BNIP3L interact with LC3 through LIR motifs in their N-terminal region and provoke subsequent mitophagy. Moreover, BNIP3 and BNIP3L compete with Beclin-1 to bind to BCL2 and BCL-XL through their intrinsic BH3 atypical domain, resulting in disruption of the BCL2/BCL-XL-Beclin-1 complex and release of Beclin-1, which is required for autophagic activity [57]. BNIP3 or BNIP3L deficiency was found to restrain ischemia-related mitophagy, demonstrating their crucial role in mitochondrial hypoxic adaptation [58, 59]. BNIP3 has been shown to be protective in renal IRI [59] and contrast-induced AKI [49] by enhancing mitophagy, while BNIP3L has a similar role in the ischemic brain [60] and in tumors in the hypoxic niche [61]. Apparently, BNIP3, BNIP3L and other receptors have overlapping and redundant functions in mitophagy, and their functional redundancy may ensure the removal of aberrant mitochondria [47].

In the ubiquitin-dependent pathway of mitophagy, the OMM ubiquitination cascade occurs on damaged and depolarized mitochondria. The decrease in the membrane potential (Δψm) of damaged mitochondria results in the stabilization of PINK1 on the OMM, where it phosphorylates ubiquitin at S65. Phosphorylated ubiquitin is capable of recruiting Parkin rapidly from the cytosol to the OMM, where Parkin is also phosphorylated by PINK1 in its ubiquitin-like domain and thus activated as an E3 ubiquitin ligase [62, 63]. Activated Parkin ubiquitinates multiple OMM proteins, and the resulting ubiquitin chains serve as autophagic signals that are recognized and bound by autophagic adaptors, e.g., p62 (SQSTM1) [64]. The adaptor molecules further associate with the phagophore via LC3, eventually leading to autophagosome formation. HIF has also been implicated in PINK1/Parkin-mediated mitophagy. It was shown that HIF-1 promoted the activation of PINK1 and Parkin and thus mitophagy in ovarian granulosa cells, but the detailed mechanism has not been identified [65]. In addition, a recent study in hepatocellular carcinoma identified HIF-1 as a regulator of PINK1 and mitophagy by promoting STOML2-mediated mitochondrial PINK1 stabilization [66], but it is not known whether this mechanism is present in other tissues. At present, although the protective effect of PINK1/Parkin-associated turnover of damaged mitochondria has been observed in AKI or ischemic preconditioning, it is unclear whether HIF is involved in the process [67–69].

The crosstalk between the two mitophagic pathways described above has been observed. BNIP3 is able to facilitate Parkin translocation to mitochondria in cardiomyocytes [70]. It also inhibits the proteolytic cleavage of PINK1 and causes an increase in the intact form of PINK1 on the OMM, leading to increased Parkin recruitment and mitophagy [71]. The importance of BNIP3 in mitophagic regulation has been further shown in animal studies. In contrast-induced AKI, deficiencies in BNIP3, PINK1 and Parkin were all found to exacerbate renal injury, and BNIP3 deficiency caused more severe injury than the other two [49, 69].

HIF-1 mediates transcriptional activation of heme oxygenase 1 (HO-1, HMOX1) [72]. HO-1 has a general antioxidant effect in tissue injuries, including AKI, by degrading the pro-oxidant heme [73]. Studies have found that HO-1 is also an essential player in mitophagy and mitochondrial quality control and protects cells from oxidative injury [74–77]. The enhanced mitophagic activity mediated by HO-1 has been linked with several downstream factors that are involved in mitochondrial quality control, as it is capable of increasing the protein expression of Parkin, PINK1, and the mitochondrial fusion mediators Mfn1/2 and OPA1 while inhibiting the expression of the fission-related proteins Fis1 and Drp1 [76, 77].

In conclusion, HIF might promote mitophagy induction in hypoxia by transcriptionally activating the mitophagy factors BNIP3 and BNIP3L; moreover, HIF is also involved in other mitophagic processes by regulating PINK1/Parkin and HO-1.

### HIF regulates mitochondrial redox homeostasis

Mitochondria are the major intracellular producers of ROS in most cell types. Mitochondrial ROS are inevitably produced as a byproduct of oxidative metabolism. ROS generation accounts for diverse cellular injuries, although it is a fundamental mitochondrial function that orchestrates many signaling pathways, including those in inflammation and autophagy [78]. There are up to 16 sites in mitochondria that possess the ability to produce ROS, 12 of which are associated with fuel oxidation and the electron transfer pathways in OXPHOS [79]. Mitochondrial ROS mainly include superoxide and hydrogen peroxide, whereas superoxide is converted rapidly to hydrogen peroxide; therefore, the latter is the major ROS released by mitochondria into the cell. Hypoxia increases mitochondrial ROS production. Mechanistically, the hypoxia-induced decrease in complex IV activity slows electron transfer along the ETC, resulting in an increase in electron transfer to molecular oxygen (O_2_) to produce more superoxide [17]. However, ROS production in isolated mitochondria decreases as the oxygen partial pressure drops, suggesting that mitochondrial ROS generation in hypoxia is not intrinsic to mitochondria alone and requires extramitochondrial factors [80, 81]. NADPH oxidase and NO have been identified to be involved in hypoxic ROS generation, but more studies are needed to fully unravel the mechanisms [80].

ROS are increased in dysfunctional/damaged mitochondria [82], which can result from prolonged mPTP opening. The increase in ROS release that is associated with maladaptive mPTP opening could induce a ROS burst propagating from one mitochondrion to another via so-called ROS-induced ROS release [80]. Damaged mitochondria also release components (e.g., mtDNA) that act as damage-associated molecular patterns to induce an inflammatory response, which could further aggravate oxidative stress [83]. Mitophagy eliminates damaged mitochondria and functions as an early response to excessive ROS; therefore, mitophagic activity is vital for a cell to maintain cellular redox equilibrium and prevent excessive ROS-induced injury [53, 84, 85].

Mitochondrial ROS are increased in many injuries and facilitate many pathological processes [86]. Taking IRI as an example, the pathogenic mechanism is multifactorial and not fully understood; however, plentiful evidence indicates that the ROS generated by mitochondria play a critical role in the initiation of cell death [87]. ROS are overproduced in both the ischemia and reperfusion stages [88]. Nevertheless, a burst of ROS generation occurs at the onset of reperfusion when oxygen is reintroduced to ischemic tissues [87]. Mitochondria are the major contributors to this hazardous burst by a process comprising calcium overload and mPTP opening [88]. Sustained mPTP formation could cause Δψm collapse and mitochondrial membrane rupture, leading to a positive feedback loop involving ROS release and inflammation. Mitochondria-targeted antioxidants provide improved protection compared with conventional antioxidants, further illustrating the essential role of mitochondrial ROS in IRI [89].

HIF is activated in hypoxia and acts as a metabolic and other regulator to support anaerobic adaptation. ROS also function as HIF-1 activators by both transcriptional activation and PHD activity regulation [90]. HIF activation in turn feeds back on mitochondrial function. As already discussed above, HIF suppresses ROS production in pathologic conditions by limiting mitochondrial oxidation and reprogramming metabolic homeostasis or by clearing damaged mitochondria and improving mitochondrial quality control. Although HIF and its several main downstream factors have the ability to decrease Δψm levels, HIF activation has also been shown to protect the integrity of ΔΨm under hypoxia-related oxidative stress by many studies [21, 36, 37].

It is presumed that a reduction in mitochondrial mass by HIF activation is responsible for the decr4-1ction in oxidative injuries. Mitochondrial turnover by mitophagy not only represents a mechanism for quality control but also reduces mitochondrial mass. Moreover, HIF-1 has been shown to induce a reduction in mitochondrial biogenesis by suppressing C-MYC/PGC-1α [91].

In addition to mitophagy, mitochondrial quality control also involves mitochondrial dynamics organization. Excessive mitochondrial fission is associated with oxidative stress, ΔΨm reduction and apoptosis activation, whereas mitochondrial fusion may protect cells against stress by limiting excessive fission [85]. It was reported that hypoxia-induced mitochondrial fission was essentially independent of HIF-1 [92]. In contrast, other studies have shown that HIF-1 promoted mitochondrial fusion while downregulating fission through HO-1, thereby lessening mitochondrial fragmentation and ROS production [36, 77].

The inner membrane cytochrome c oxidase (COX) complex catalyzes the vital step of electron transport to O_2_. By transcriptionally prompting the expression of COX4-2 and LON (the peptidase degrading COX4-1), HIF-1 switches the COX composition from COX4-1 to COX4-2, resulting in less ROS production without impairing ATP production. However, this function of HIF to induce the favorable COX4 isoform is tissue-dependent and has not been found in the kidney [93].

Furthermore, HIF can boost the activity of the cellular antioxidant defense system. Glutathione (GSH), a tripeptide synthesized from cysteine, glutamate and glycine, is the major molecule that defends against ROS-related injury. HIF-1 promotes GSH synthesis by upregulating the expression of SLC7A11 and GCLM [94]. SLC7A11 is an essential subunit of the cystine transporter System X_C_^−^, which mediates cystine uptake. GCLM (glutamate-cysteine ligase modifier subunit) is an integral part of γ-glutamylcysteine synthetase, which is a rate-limiting enzyme catalyzing the first step of glutathione (GSH) synthesis. Moreover, HIF-1 increases the expression of glutaminase, thereby promoting the production of glutamate and thus GSH [95]. NADPH is essential for the recycling of GSH and is thus involved in redox defense. HIF was shown to induce the overexpression of SHMT2 [96] and PHGDH [97] to support serine metabolism-related NADPH synthesis, thereby maintaining mitochondrial redox homeostasis in hypoxia.

NRF2 has been recently recognized as a master transcription factor that activates antioxidant enzymes [98]. Accumulating evidence has shown considerable and complex crosstalk between the HIF-1 and NRF2 signaling pathways. They have a number of common downstream components and effector molecules and may work in concert to reinforce each other in some situations [99]. Some studies have shown that the HIF PHD inhibitor FG-4592 counterbalances oxidative stress via NRF2 activation [100, 101]. However, there have also been studies showing that HIF-1 and NRF2 have inhibitory effects on each other. Therefore, more mechanistic studies are needed to better understand their interactions. In the kidney, the stress-responsive transcription factor FoxO3 was found to reduce oxidative stress and promote autophagy in tubular cells in AKI, thereby attenuating CKD development following AKI. Interestingly, FoxO3 activation was induced by hypoxia and HIF-1 in AKI [102]. It is not known whether NRF2 is involved in the activation of FoxO3 in the AKI-CKD transition.

## Involvement of mitochondrial disorder and ferroptosis in AKI

Ferroptosis is a type of regulated cell death that is usually accompanied by a large amount of iron accumulation and lipid peroxidation in cells. The collapse of cellular redox homeostasis that occurs when the intracellular production of lipid ROS exceeds the capacity of the lipophilic antioxidant system provides the driving force for this cell death process. The cyst(e)ine/glutathione peroxidase 4 (GPX4)/GSH axis is recognized as the mainstay in reducing lipid peroxidation. There have been a number of excellent reviews on the topic of ferroptosis in recent years [103–107]. Here, we mainly discuss its relationship with mitochondrial ROS in AKI.

### Role of ferroptosis and ferroptosis-targeting interventions in different types of AKI

Although apoptosis has been identified as a form of tubular cell death in AKI for more than two decades, it is now considered that apoptosis is not the driving mechanism of AKI, as apoptosis interference appears to be clinically irrelevant in renal injury [108]. Studies have shown that regulated necrosis, including ferroptosis and necroptosis, plays a central pathophysiological role in AKI, especially in IRI, which provides a new avenue for AKI prevention and treatment [108, 109]. Compelling evidence has proven that ferroptosis represents a major form of cell death in AKI [108–112].

Renal IRI caused by clinical events such as circulatory “shock” and cardiac surgery is a common cause of AKI and contributes significantly to the morbidity and mortality of AKI. In the ischemia and hypoxia phase, tissue aerobic metabolism is jeopardized, which may finally lead to ATP insufficiency, cellular acidosis and edema. Reperfusion restores oxygen supply but concomitantly causes ROS production and oxidative damage in the cells [87]. Linkermann et al. reported that the small molecule ferrostatin-1 or its more stable derivative 16–86, which blocks lipid peroxidation to combat ferroptosis, was protective in a murine model of severe IRI [113]. They also demonstrated that ferroptosis occurs in a synchronized manner in renal tubules and eventually causes the failure of entire functional units. Another antioxidant, namely, liproxstatin-1, has a similar effect and mechanism as ferrostatin-1 and protects against renal IRI [114, 115]. The lipid metabolic enzyme ACSL4 promotes ferroptosis by increasing the PUFA content in the phospholipids of the plasma membrane. PUFAs are susceptible to oxidation reactions, resulting in ferroptosis. Müller et al. found that ACSL4 is upregulated in the kidneys of an IRI animal model and posttransplantation patients and that ACSL4 deficiency confers protection against ferroptosis [116]. Stoppe et al. showed that MIF has a protective effect on the kidney in both an IRI animal model and postsurgery AKI patients by inhibiting necroptosis and ferroptosis. MIF inhibition of ferroptosis was attributed to its capability of restoring intracellular GSH and reducing oxidative stress [117]. Huang et al. found that ALR, a sulfhydryl oxidase, is indispensable for cells to defend against ferroptosis in renal IRI, and this protective role of ALR lies in its antioxidant effect through its interaction with GPX4 [118]. With single-cell RNA sequencing, Zhao et al. found that ferroptosis-related genes were mainly expressed in tubular cells after IRI; in contrast, necroptosis- and pyroptosis-associated gene expression was low in the cells [119]. They further showed that the mitochondria-targeted antioxidant XJB-5-131 could protect against renal tubular cell injury by inhibiting ferroptosis. These studies highlight the central role of mitochondrial oxidative stress, lipid peroxidation, and ferroptosis in renal IRI pathology.

AKI induced by folic acid overdose in mice recapitulates all the major processes in human AKI, which is characterized by ferroptosis and secondary immunogenicity [120]. Interestingly, ferrostatin-1, but not necroptosis-targeting agents, was capable of preserving the renal function of the mice in the study, regardless of the upregulation of the necroptosis markers MLKL and RIPK3 in the kidney. Pretreatment with FG-4592 was found to attenuate folic acid-induced renal ferroptosis via a mechanism involving NRF2 activation [101]. Rhabdomyolysis can induce AKI in patients. Studies have shown that ferroptosis plays an important role in rhabdomyolysis-induced renal damage, and pretreatment with antioxidants, including curcumin, ferrostatin-1 [121], and recombinant MIF [117], mitigates renal injury. Ferroptosis is also responsible for oxalate crystal-induced AKI [113]. With regard to cisplatin-induced AKI, although there has been evidence supporting the central pathological role for necroptosis [108], iron dysregulation has also been identified in cisplatin-induced AKI [122]. Recently, Deng et al. found a proximal tubular cell–specific enzyme, MIOX, and showed that MIOX mediates ROS production and ferroptosis, thereby contributing to cisplatin-induced AKI [123]. On the contrary, contrast-induced AKI exhibits some features of osmotic nephrosis in which tubules undergo marked apoptotic, but not necrotic, cell death [49, 108].

In addition to pharmacological evidence, recent studies have further demonstrated the contribution of ferroptosis to AKI via direct genetic manipulations. Ferroptosis suppressor protein 1 (FSP1, also known as AIFM2) was newly identified as an oxidoreductase that reduces coenzyme Q_10_ to a lipophilic radical-trapping antioxidant in the plasma membrane, thus acting in parallel with GPX4 to suppress phospholipid peroxidation and ferroptosis [124]. Both loss of Fsp1 and targeted manipulation of the active center of Gpx4 sensitize mice to ferroptosis in tubules in renal IRI, while the latter showed more dramatic sensitization [125]. Another study showed that inducible disruption of Gpx4 in mice caused acute tubular cell death [115]. As in this study, the kidney was the most commonly involved and may account for animal mortality; thus, it is interesting to suppose that the kidney might be the organ that is more prone to ferroptotic injury.

### Relationship between mitochondria and ferroptosis

Mitochondria are central to the initiation of the intrinsic pathway of apoptosis. Nevertheless, there are some controversies regarding their contribution to ferroptosis according to recent studies.

Various studies have shown that ferroptotic cell death is accompanied by morphological and functional changes in mitochondria. Currently, the identified morphological features of ferroptotic cells mainly include mitochondrial size reduction, increased bilayer membrane density, disappearance of the mitochondrial cristae, and OMM disruption, suggesting extensive involvement of mitochondria in the ferroptotic process [107].

Functionally, mitochondrial energetic metabolism and ferroptosis closely interact with each other. In a landmark study, Gao et al. found that both cysteine deprivation-induced (CDI, including cysteine starvation and treatment with the SLC7A11 inhibitor erastin) and GPX4 inhibitor RSL3-induced ferroptosis led to Δψm hyperpolarization (which eventually collapses), while the mitochondrial uncoupler CCCP inhibited the process [126]. CCCP also prevented ROS generation and ferroptosis in cysteine deprivation but not in GPX4 inhibition-induced ferroptosis. The researchers further revealed that both the mitochondrial TCA cycle and ETC activity are required for potent CDI ferroptosis using fumarate hydratase (FH) mutant cells and ETC inhibitors, respectively, suggesting that mitochondria are actively involved in ferroptosis by fueling metabolism and ROS generation. Knockdown of dihydrolipoamide dehydrogenase (DLD), a component of the α-ketoglutarate dehydrogenase complex, could also block Δψm hyperpolarization, the increase in lipid ROS and ferroptosis in cysteine deprivation [127].

Voltage-dependent anion channels (VDACs) on the OMM are the principal sites for the exchange of a variety of metabolites between mitochondria and the cytosol. The ferroptotic inducer erastin not only inhibits X_C_^−^ but also directly induces the opening of VDAC2/3 [128], which leads to increased mitochondrial metabolism (by augmented entry of substrates into mitochondria), mitochondrial hyperpolarization and ROS formation [129]. Erastin could induce ferroptosis in cystine deprivation medium, suggesting the key role of VDAC opening [130]. Another study showed that phospholipid peroxidation-related VDAC2 carbonylation plays an important role in RSL3-induced ferroptosis [131].

Iron overload sensitizes cells to ferroptosis in vitro and in vivo as iron is engaged in free radical formation and propagation of lipid peroxidation [132]. Lipids undergo spontaneous peroxidation in the presence of hydroxyl radicals generated from the Fenton reaction of redox-active Fe^2+^ and hydrogen peroxide [133]. Mitochondria are the major site for iron metabolism and homeostasis [134]. Mitochondrial free iron accumulation is assumed to contribute to ferroptosis. This is because mitochondrial iron overload has been detected in multiple ferroptosis models [127, 135, 136]; moreover, cellular iron overload has been shown to induce mitochondrial ROS generation and damage [137]. Mitochondrial ferritin (FtMt) is an iron-storage protein that can oxidize Fe^2+^ to catalytically inactive Fe^3+^ and maintain free iron homeostasis in mitochondria. Overexpression of FtMt counteracted erastin-induced ROS and ferroptosis both in vitro and in vivo [138]. Proteins involved in mitochondrial and cytosolic iron exchange play a crucial role in regulating erastin-induced ferroptosis: CDGSH iron sulfur domain 1 (CISD1, also termed mitoNEET) can inhibit ferroptosis [135], while the BRD7-P53-SLC25A28 axis promotes ferroptosis induction by facilitating mitochondrial iron accumulation and ETC hyperfunction [139]. Frataxin is an iron chaperone that drives the assembly of the Fe-S cluster in mitochondria, while it has recently been identified as a key regulator of ferroptosis by modulating mitochondrial iron homeostasis [140]. The recessive disorder Friedreich’s ataxia results from frataxin insufficiency. Ferroptotic features, such as mitochondrial iron accumulation, increased lipid peroxidation and oxidative stress, have been observed in Friedreich’s ataxia for a long time, making anti-ferroptosis drugs promising in the treatment of the disease [141].

BH3-interacting domain death agonist (BID), a proapoptotic protein of the Bcl-2 family, is translocated to mitochondria, where it mediates ROS production, loss of Δψm and mitochondrial integrity, and ferroptotic cell death in erastin-treated neuronal cells [142]. Ferrostatin-1 could prevent BID translocation to the mitochondria in erastin treatment but failed to protect cells from overexpression of the activated form of BID, suggesting that BID is a sensor for iron-dependent generation of oxidative stress and that its transactivation serves as the final execution step in mitochondrial cell death.

Overall, the most direct evidence that supports a role of mitochondria in ferroptosis is from mitochondrial targeting interventions. However, cells depleted of mitochondria [143] or mtDNA [144] are still sensitive to ferroptosis inducers, indicating that mitochondria can be dispensable for ferroptosis execution, at least under certain conditions. Paradoxically, several mitochondrial proteins, such as FH and DLD, as mentioned above, are required for ferroptosis induction. The mitochondria-targeted ROS scavengers XJB-5-131 [145] and mitoquinone [146] can also prevent ferroptotic cell death in various cell types. One explanation for the discrepancy is that the role of mitochondria in ferroptosis could be cellular context dependent. In particular, metabolism, including lipid ROS generation, in mitochondria-depleted cells was fundamentally rewired, which might have affected ferroptosis execution [126]. Moreover, a potential contribution of the particular conditions of experiments to the discrepancy should also be taken into account. As mentioned above, Gao et al. demonstrated the active involvement of mitochondria in CDI ferroptosis but not in RSL3-induced ferroptosis, which might be due to the most downstream role of GPX4 in ferroptosis [126]. Consistent with this, the mitochondrial depletion study by Gaschler et al. also showed a significant decrease in ferroptosis sensitivity after mitophagy in CDI ferroptosis [143]. In conclusion, at present, although there are still some controversies concerning the details of the mitochondrial involvement in the ferroptotic process, it has been well established that abnormalities in mitochondrial homeostasis are sufficient and, in many cases, indispensable for ferroptosis induction.

## Regulation of mitochondria and ferroptosis by HIF/PHD inhibitors in AKI

### Protection of HIF and PHD inhibitors against AKI

As the key regulator of oxygen homeostasis, HIF-1 has been shown to exert a protective role in ischemia-related renal injuries [147]. Compelling evidence has further proven the protective effect of HIF on renal IRI with complex mechanisms involving the regulation of ROS production and inflammation, which remain incompletely understood [148]. PHD inhibitors, such as dimethyloxaloylglycine and FG-4487, have also been explored [149, 150]. As HIF is activated in IRI, whether the beneficial effects of PHD inhibitors derive simply from their activation of HIF is questioned. Importantly, it has been shown that the time point for PHD inhibitor treatment is critical for the optimal effect. PHD inhibition prior to AKI was shown to ameliorate fibrosis, while inhibition in the early recovery phase of AKI did not affect the renal outcome [22].

Accordingly, PHD inhibitors should share the same rationale in AKI treatment as ischemic preconditioning (IPC), which is effective in preventing renal IRI in animal models [151]. IPC can induce prolonged HIF stabilization by ROS-related PHD inactivation [152]. HIF-1 is a central mediator of IPC-induced protection, as shown in the heart of HIF-1 knockout mice [153] and in the kidney of mice treated with a HIF-1α inhibitor [154]. Studies have demonstrated that IPC can promote a number of adaptive changes involving metabolism, inflammatory responses, oxidative stress and angiogenesis and that some of the changes are associated with HIF-mediated transcriptional alterations [155]. Studies with the heart have shown that the protective effects of IPC depend on its ability to reduce mPTP opening and attenuate mitochondrial dysfunction in reperfusion [156, 157]. In renal IRI, mitophagy induction, which clears damaged mitochondria, was recently shown to be obligatory for the protective effect of IPC [67]. Both receptor-dependent and receptor-independent mitophagy may be involved in the IPC process, as evidenced by a study in which proximal tubule-specific FUNDC1 knockout abolished the renal protective effect of IPC [158] and another study showing the requirement of PINK1 for the renal protective effect of IPC [67].

The beneficial effects of HIF on cisplatin-induced AKI have also been shown [159]. The PHD inhibitor FG-4592 and PHD2 knockout both markedly ameliorated cisplatin-induced tubular injury and oxidative stress [160, 161]. In folic acid-induced AKI, FG-4592 was found to attenuate oxidative stress and renal damage via Nrf2 nuclear translocation and upregulation of downstream proteins, including HO-1, GPX4 and SLC7A11 [101]. Rhabdomyolysis-induced AKI can also be alleviated by HIF activation induced by VHL knockout in renal tubules, and an improvement in glucose metabolism may be implicated in the process [162].

### Perspectives of the relationship between HIF activation and ferroptosis in AKI

Very few studies have investigated the association between HIF activation and ferroptosis at present. In 2020, Li et al. found that the PHD inhibitor FG-4592 attenuated ferroptosis in folic acid-induced AKI by promoting the Akt/GSK-3β/NRF2 pathway [101]. Ferritinophagy is the process of selective degradation of ferritin, which can promote ferroptosis by increasing the “labile iron pool”. HIF-1α was shown to decrease ferritinophagy via inhibition of autophagosome formation, thus protecting osteoclasts from ferroptosis [163]. Iron chelators, such as deferoxamine, can stabilize HIF and have been shown to inhibit ferroptosis, but whether the two effects are related is unknown [164]. In contrast, in clear-cell carcinomas, HIF-2α activation was found to increase the vulnerability of cells to ferroptosis by enriching polyunsaturated lipids [165].

**Fig. 3:**
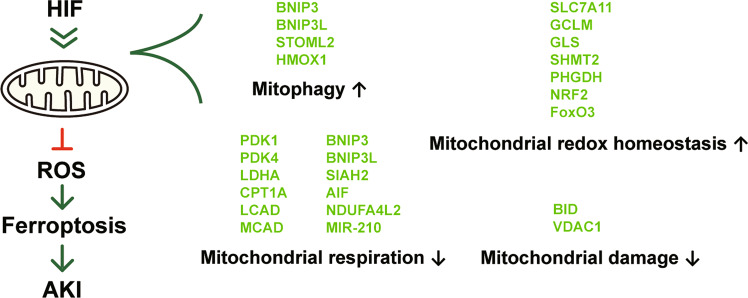
Proposed mechanisms by which HIF can ameliorate ferroptosis in AKI. HIF regulates a series of genes that affect mitochondrial function. By promoting mitophagy and limiting mitochondrial aerobic respiration, the production of ROS and the active involvement of mitochondria in the ferroptotic process could be restrained. In addition, HIF maintains mitochondrial redox homeostasis and has protective effects against mitochondrial damage. The overall effects of HIF on mitochondria rationally explain how it protects against AKI, which was proven to be a pathological process centrally involving ferroptosis. The molecules directly regulated by HIF are shown in lime color.

Considering that ferroptosis has a central role in most forms of AKI and that HIF PHD inhibitors have been proven to exert protective effects in AKI, we propose that PHD inhibitors may have an anti-ferroptosis effect in AKI treatment. The PHD inhibitor FG-4592 was shown to decrease ferroptosis in a folic acid-induced injury model, but the mechanism of protection is not comprehensively understood [101]. From our perspective, HIF may function through a mechanism that is more complex than that of NRF2 to antagonize ferroptosis. Based on the current understanding of HIF regulation, we propose the following mechanisms that may explain the protection provided by HIF against ferroptosis in AKI (Fig. 3).

First, HIF may suppress ferroptosis in AKI by promoting mitophagy. By eliminating damaged mitochondria, mitophagy was shown to be indispensable for the protective effect of IPC in AKI. Mitophagy might contribute to ferroptosis inhibition by decreasing ROS production, which is exaggerated in damaged mitochondria. In addition, mitochondrial mass reduction by mitophagy and restrained biogenesis may also limit ROS production and ferroptosis induction, especially in the reperfusion stage of IRI.

Second, HIF may suppress ferroptosis in AKI by decreasing mitochondrial respiration. Studies have shown that mitochondrial metabolism, including both the TCA cycle and ETC activity, can prompt ferroptosis under specific conditions. As discussed above, HIF activation represses overall mitochondrial metabolism, including the TCA cycle and ETC activity. The mitochondrial uncoupler CCCP is capable of preventing ferroptosis and protecting against renal IRI as a hypoxia inducer [67], suggesting that PHD inhibitors may have a similar effect. However, it remains undetermined whether ferroptosis in AKI involves mitochondrial hyperactivity in renal cells.

Third, HIF may suppress ferroptosis in AKI by maintaining mitochondrial redox homeostasis. Studies have already found that HIF is able to suppress ferroptosis by upregulating NRF2 and downstream proteins such as HO-1 and GPX4 [101]. HIF also regulates redox balance by a variety of pathways, some of which may lead to ferroptosis inhibition. For instance, HIF-1 promotes GSH synthesis, which is the main antioxidant against ferroptosis [94].

Finally, HIF may suppress ferroptosis in AKI by limiting mitochondrial damage. HIF activation is known to relieve mitochondrial damage in IRI by multiple mechanisms. HIF and ferroptosis may be linked by BID because BID translocation induces mitochondrial damage and represents a “point of no return” in mitochondrial cell death [142]. HIF-1α transcriptionally downregulates BID, thereby enhancing the suppression of apoptosis in cancer cells [166]. Moreover, OMM-linked HIF-1α was shown to protect mitochondria by regulating VDAC1, Δψm and mtDNA transcription, as mentioned above [37, 45].

It should be pointed out that PHD inhibitors may inhibit ferroptosis by a mechanism beyond HIF stabilization. HIF PHDs are also known to hydroxylate substrates, e.g., ATF4, which has been found to be involved in GPX4 expression and ferroptosis regulation [167, 168]. Targets other than PHDs should also be considered with the use of PHD inhibitors [169].

## Final remarks

In the past decade, the pivotal role of ferroptosis in pathogenesis has been recognized in most forms of AKI, and ferroptosis-targeting interventions have been proven to effectively protect the kidney in animal models of AKI. In consideration of the established notion that preactivation of HIF could protect the kidney from AKI, especially renal IRI, there seems to be a missing link between HIF and ferroptosis in the kidney. Proximal tubular cells are particularly susceptible to the harmful effects of mitochondrial damage, as they are highly energy demanding and deeply depend on oxidative mitochondrial metabolism. Apparently, proximal tubular cells should be prone to mitochondrial ROS-related ferroptosis, as has already been shown [115, 125].

In this article, we have reviewed and analyzed the current understanding of the interplay between HIF, mitochondria and ferroptosis. Based on this information, we propose that HIF activation is involved in the regulation of ferroptosis in AKI by multiple potential mechanisms and that PHD inhibitors may represent a new therapy for patients with AKI. Apparently, more studies are needed to confirm these speculations and to address the existing paradoxical observations concerning the relationship between HIF, mitochondrial function, and ferroptosis.

## Data Availability

All relevant data are included in this manuscript.
